# Expression of Luteinizing Hormone-Releasing Hormone (LHRH) and Type-I LHRH Receptor in Transitional Cell Carcinoma Type of Human Bladder Cancer

**DOI:** 10.3390/molecules26051253

**Published:** 2021-02-26

**Authors:** Zsuzsanna Szabó, Balázs Dezső, Klára Fodor, Krisztián Szegedi, Tibor Flaskó, Erzsébet Szabó, Gábor Oláh, Éva Sipos, Nikoletta Dobos, János Gardi, Andrew V. Schally, Gábor Halmos

**Affiliations:** 1 Department of Biopharmacy, Faculty of Pharmacy, University of Debrecen, 4032 Debrecen, Hungary; szabo.zsuzsanna@pharm.unideb.hu (Z.S.); fodor.klara@pharm.unideb.hu (K.F.); erzsebet.szabo@med.unideb.hu (E.S.); olah.gabor@pharm.unideb.hu (G.O.); sipos.eva@pharm.unideb.hu (É.S.); dobos.nikoletta@pharm.unideb.hu (N.D.); 2 Department of Pathology, Clinical Center and Section of Dental Microbiology and Oral Pathology, Faculty of Dentistry, University of Debrecen, 4032 Debrecen, Hungary; bdezso@med.unideb.hu; 3 Department of Urology, Clinical Center, University of Debrecen, 4032 Debrecen, Hungary; szegedi.krisztian@med.unideb.hu (K.S.); flash@dote.hu (T.F.); 4 Doctoral School of Pharmaceutical Sciences, University of Debrecen, 4032 Debrecen, Hungary; 5 Department of Medicine, Faculty of Medicine, University of Szeged, 6720 Szeged, Hungary; gardi.janos@med.u-szeged.hu; 6 Veterans Affairs Medical Center, Endocrine, Polypeptide and Cancer Institute, Miami, FL 33101, USA; andrew.schally@va.gov; 7 Sylvester Comprehensive Cancer Center, Department of Medicine, Department of Pathology, Divisions of Hematology Oncology and Endocrinology, Miller School of Medicine, University of Miami, Miami, FL 33101, USA

**Keywords:** LHRH receptor, targeted therapy, bladder cancer, radioligand binding assay, immunohistochemistry, Western Blot, RT-PCR

## Abstract

Bladder cancer (BC) is the tenth most frequently detected cancer in both sexes. Type-I luteinizing hormone-releasing hormone (LHRH) receptor (LHRH-R-I) is expressed not only in the pituitary, but also in several types of cancer disease. There are few data about LHRH-R-I expression in human BC. This study aimed to investigate the expression of LHRH and LHRH-R-I in the transitional cell carcinoma (TCC) type of human BC. RNA was extracted from 24 human bladder tumor specimens and three BC cell lines. RT-PCR was performed to detect mRNA for LHRH and LHRH-R-I. The protein of LHRH-R-I was further studied by immunohistochemistry (IHC), ligand competition assay, and Western Blot. PCR products of LHRH were found in 19 of 24 (79%) specimens and mRNA of LHRH-R-I was detected in 20 of 24 specimens (83%). Positive immunostaining for LHRH-R-I with different expression intensity was found in all samples examined, showing negative correlation with TCC grade. Radioligand binding studies also showed the presence of specific LHRH-R-I and high affinity binding of LHRH analogs. The high incidence of LHRH-R in BC suggests that it could serve as a molecular target for therapy of human BC with cytotoxic LHRH analogs or modern powerful antagonistic analogs of LHRH.

## 1. Introduction

Cancers of the genitourinary system include those of the kidneys, bladder, and prostate, as well as less common cancers such as those of the urethra and ureters [1,2,3,4]. Bladder cancer is the 10th most common cancer worldwide and it is the second most common malignancy of the genitourinary tract after prostate cancer. It is rarely diagnosed in individuals younger than 40 years [1,2,4]. Yearly almost 400,000 new cases of urinary bladder cancer are diagnosed and more than 150,000 people die worldwide of the disease. The most common type of bladder cancer is the transitional cell carcinoma (95.7%) and most tumors are of low malignant potential grade (39.7%) [4,5,6]. Despite its chemosensitivity, bladder cancer has high recurrence rate, nearly 50% and has the highest lifetime treatment cost per patient of all cancers [2,7]. Systemic chemotherapy and immunotherapy result in partial response and only few cases show a complete response to this kind of combination therapy. These therapeutic approaches are accompanied by severe side effects and consequently by a low quality of life, therefore, new treatment strategies are urgently needed [5,8].

Luteinizing hormone-releasing hormone (LHRH), also known as gonadotropin releasing hormone (GnRH), is a decapeptide (pGlu-His-Trp-Ser-Tyr-Gly-Leu-Arg-Pro-Gly-NH_2_) [9]. It is the pivotal hormone of the hypothalamo-pituitary gonadal axis. Most vertebrate species possess at least two, and usually three forms of LHRH, which differ in their amino acid sequences, localizations, and embryonic origins [9]. In the human genome only LHRH-I (mammalian LHRH) and LHRH-II (chicken LHRH) genes have been identified [10]. Although LHRH-II has been found in tumors such as breast carcinoma, the functional receptor for LHRH-II is absent in humans [11,12].

Type-I LHRH receptor (LHRH-R-I) belongs to the G protein-coupled receptor (GPCRs) family which contains a characteristic seven-transmembrane (7TM)-domain structure [13,14]. Unlike other members of the GPCR family, the LHRH-R-I lacks the entire carboxyl-terminal tail [9,14,15]. Expression of LHRH-R-I has been demonstrated in normal and malignant tissues [9,15]. Based on their ligand binding properties, LHRH receptors appear to be similar in the human pituitary and normal human extrapituitary tissues. However, in human cancers, their signaling pathways are different [16]. LHRH receptors have been found on breast, prostatic, ovarian, endometrial, pancreatic cancers, renal cell carcinoma, and uveal melanoma [9,12,15,17,18,19,20]. LHRH receptors were also found in human benign prostatic hyperplasia [21,22]. The expression of peptide hormone receptors at different levels in malignant and normal tissues provides the opportunity for targeted therapy [9,12,15,18,23].

The human bladder epithelia also produce luteinizing hormone-releasing hormone and LHRH receptors in both normal and malignant tissues; however, their role is not completely understood [24]. The effects of the androgen regulation system and LHRH agonist on bladder carcinogenesis have been investigated in animals [10]. Previous studies suggested that the suppression of testicular androgen can inhibit bladder carcinogenesis. Castration suppressed bladder carcinogenesis, LHRH agonist promoted bladder carcinogenesis, and flutamide suppressed the LHRH-induced promotion of carcinogenesis [25].

LHRH receptors on tumor cells can mediate direct effects of LHRH analogs [9,15,23]. A new class of antitumor drugs has been developed based on LHRH analogs bearing cytotoxic radicals, such as doxorubicin and its derivative 2-pyrrolino-doxorubicin [9,15,18,26]. The cytotoxic LHRH analogs AN-152 and AN-207 are less toxic and much more effective in vivo than their respective radicals in inhibiting tumor growth in LHRH-receptor-positive models of human ovarian, mammary, endometrial, and prostatic cancers xenografted into nude mice [9,15,27,28]. In the past 25 years, several thousand of LHRH analogs have been synthesized, and many analogs were shown to have important clinical applications in gynecology, oncology, and urology [9,12,15,18,27,28].

The diagnosis and follow-up of patients require expensive invasive methods; therefore, bladder cancer continues to be one of the most costly malignancies to treat. The increasing trend in incidence of bladder cancer in recent decades, along with the lack of adequate knowledge on this malignancy and about its therapy, emphasize the importance to research on the expression of LHRH receptors as potential molecular targets in urological tissues. The incomplete data about LHRH receptors in bladder cancer prompted us to investigate the expression pattern of LHRH-I and LHRH-R-I in human bladder tumor specimens and in three human bladder cancer cell lines (RT-112, UMUC3, and TCCSUP). The expression of mRNA for LHRH-I and LHRH-R-I was investigated by RT-PCR in surgical specimens of bladder cancer and in three bladder cancer cell lines, using gene specific primers. The presence and binding characteristics of LHRH receptor protein were further studied by immunohistochemistry, Western Blot, and ligand competition assay.

## 2. Results

### 2.1. Expression of mRNA for LHRH-I and LHRH-R-I in Human Bladder Cancer Tissue Samples and in Human Bladder Cancer Cell Lines

**Figure 1:**
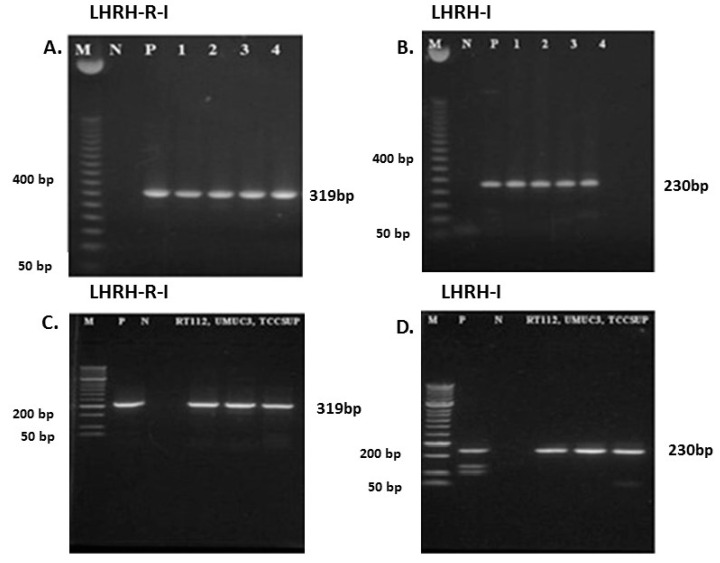
Representative RT-PCR analysis of mRNA for LHRH-I and LHRH-R-I in human bladder tumor specimens and three bladder cancer cell lines. (**A,C**) The PCR products were of the expected size of 319 bp for LHRH-R-I; (**B,D**) The PCR products were of the expected size of 230 bp for LHRH-I). Lane M, molecular marker (50-bp DNA ladder); Lane P, positive control (human pituitary tissue); Lane N, no template control; Lanes 1–4 (**A**,**B**) representative human bladder cancer specimens; Panels (**C**,**D**) representative human bladder cancer cell lines (RT-112, UMUC3, and TCCSUP).

In order to examine the expression pattern of LHRH-I and mRNA for LHRH-R-I in human bladder cancer tissues and in human bladder cancer cell lines, RT-PCR was performed on all the samples collected for the study. Template-free and reverse transcriptase-free controls excluded non-specific amplification and DNA contamination, respectively. PCR amplification with specific primers for β-actin produced a single product in every sample, confirming no RNA degradation in the samples. As a positive control, human pituitary samples were used. PCR amplification derived from the bladder tumoral tissues and cell lines using the oligonucleotide primers for human LHRH receptor yielded the expected 319 bp for LHRH receptors (Figure 1A,C).

In 20 (83%) of 24 specimens examined, RT of RNA followed by PCR amplification with specific primers produced a fragment of the expected size (319 bp) for LHRH receptors (Table 1). Our results clearly show that mRNA for LHRH-R-I was also expressed in those three human bladder cancer cell lines (RT-112, UMUC3 and TCCSUP) examined (Figure 1C).

In order to examine the mRNA expression of LHRH ligand, we also performed RT-PCR analyses on 24 samples of human bladder cancer tissues obtained from the Department of Urology. In 19 of 24 specimens examined (79%), RT of RNA followed by PCR amplification with specific primers produced a fragment of the expected size (230 bp) for LHRH (Figure 1B) (Table 1). The mRNA both for LHRH and LHRH receptor was expressed in 15 tumor samples (62.5%). The mRNA for LHRH ligand was also expressed in three human bladder cancer cell lines examined (Figure 1D).

### 2.2. Immunohistochemistry of LHRH Receptor in Human Bladder Cancer Tissue

**Figure 2:**
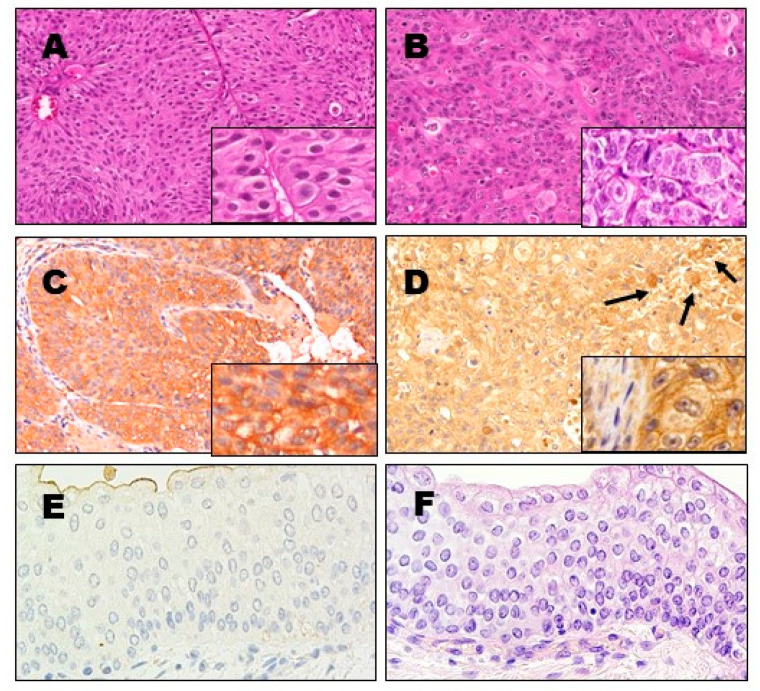
Expression levels of LHRH receptor protein in surgically removed human TCC bladder cancer cases detected by immunohistochemistry (IHC). (**A**,**B**) Hematoxylin–eosin stained sections of representative samples. (**C**,**D**) The corresponding IHC images for LHRH receptor expression. (**A**) shows a papillary TCC, G1 (low grade) expressing (**C**) cytoplasmic and membranous (inset) LHRH receptor protein, predominantly with score 3+ intensities. (**B**) shows an invasive TCC, G3 (high grade) with cellular pleomorphism harboring (**D**) predominantly low intensity (score 1+) but irregular expression levels for the LHRH receptor protein with a few 2+ positive cells (arrows). Insets of images are to demonstrate the cellular details and IHC staining patterns, respectively. (**E**,**F**) Images of normal urothelial mucosa showing no immunohistochemically detectable LHRH receptor protein expression (**E**) albeit trace apical positivity can be recognized (light brown staining at upper left), most likely reflecting background staining. (**F**) is a corresponding hematoxylin–eosin stained image of the normal urothelium for cellular detail. (*Original magnifications for images A–D: 200×; insets and images E–F: 400×).*

A total of 12 human bladder cancer specimens were obtained from primary tumors (metastases were not observed) and evaluated for LHRH receptor expression by immunohistochemistry. Positive staining for LHRH receptors was found in all samples examined, independently from transitional cell carcinoma (TCC) grade, but with different intensity of expression (Figure 2) (Table 2).

The majority of TCCs expressed the LHRH receptor at a moderate level and the majority of cells showed cytoplasmic or membranous LHRH-R-I expression characterized by brown granules within tumor cells. The following conclusion could be generated: the less differentiated TCC cases (Grade 3–4) showed no or weak staining (0, 1+), but the well or moderately differentiated tumors (Grade 1–2) appeared to exhibit moderate to strong LHRH-R expression (Figure 2). We prepared a Pearson analysis and our results showed that the expression of LHRH receptors is negatively correlated (r = −0.91) with the pathological grade of the cases examined. No correlation among LHRH-R-I expression and patients’ age and gender was observed. The collection of fresh, normal human bladder tissue specimens is very difficult. However, we were able to investigate 3 adjacent healthy bladder tissue samples. There was no LHRH receptor expression detected in these specimens.

### 2.3. Expression of LH-RH Receptors in Human Bladder Cancer Cell Lines In Vitro

**Figure 3:**
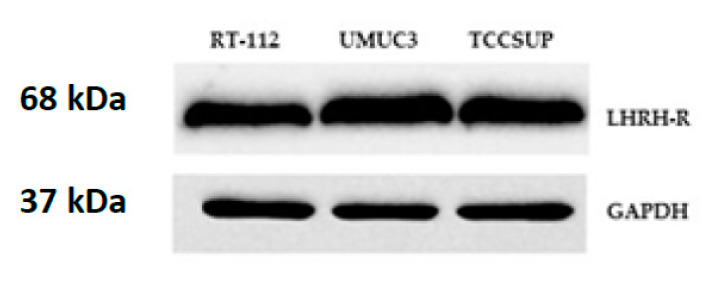
Western blot analysis of LHRH-receptor protein in human bladder cancer cell lines RT-112, UMUC3, and TCCSUP. GAPDH was used as a housekeeping protein.

The presence of LHRH receptors was investigated in RT-112, UMUC3, and TCCSUP cell lines by Western blot analysis. Our results confirmed the presence of LHRH receptors in all three bladder cancer cell lines examined and revealed a signal corresponding to a protein of ~68 kDa, which is the molecular mass of the LHRH-I receptor protein reported earlier [9,15,23]. In accordance with the receptor mRNA data, high protein expression of LHRH receptors was observed in RT-112, UMUC3, and TCCSUP cell lines by Western blot (Figure 3).

### 2.4. Radioligand Binding Studies

Using ligand competition assays, a single class of high affinity binding sites for LHRH was found in the 12 human bladder cancer specimens examined. Of the 12 cancer specimens investigated, 10 displayed LHRH receptor binding (Table 3).

**Figure 4:**
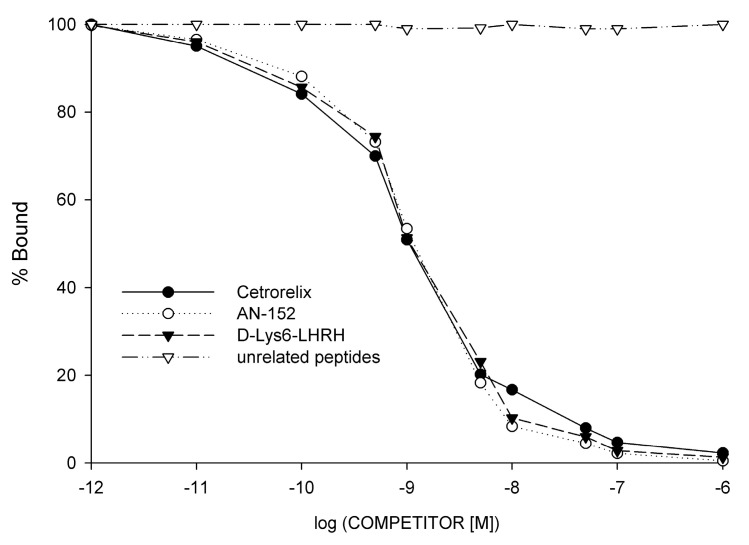
Representative displacement of [^125^I][D-Trp^6^]LHRH binding to membrane fractions of human bladder cancer specimens by increasing concentrations of LHRH antagonist Cetrorelix (●), agonist [D-Lys^6^]LHRH (▼), and cytotoxic LHRH analog AN-152 (○). Other unrelated peptides, like somatostatin, human growth hormone (hGH), growth hormone-releasing hormone (GHRH), vasoactive intestinal peptide (VIP), and epidermal growth factor (∆) did not displace the radioligand. Each point represents mean of duplicate or triplicate determinations.

Analysis of the typical displacement of radiolabeled [D-Trp^6^] LHRH and the Scatchard plots of the specific binding data in the 10 receptor positive bladder cancer specimens revealed that LHRH receptors had a mean dissociation constant (K_d_) of 4.98 nM (range, 3.61 to 6.85 nM). The mean concentration of LHRH receptors (maximal binding capacity, Bmax) was 473.09 fmol/mg membrane protein in crude membranes derived from human bladder cancer cells (range, 255.0 to 721.4 fmol/mg protein). Biochemical parameters essential to establish the identity of specific binding sites were also determined. Thus, the binding of [^125^I][D-Trp^6^]LHRH was found to be specific, reversible, time- and temperature-dependent, and linear with protein concentration in human bladder cancer samples (Figure 4). The expression of mRNA for LHRH receptors was accompanied by ligand binding in all tumor specimens examined. The binding affinity of LHRH analogs and cytotoxic LHRH analog AN-152 (AEZS-108) to membrane receptors of human bladder cancer cells expressing LHRH receptors was also investigated by ligand competition assay. Displacement of [^125^I][D-Trp^6^]LHRH as a radioligand by the unlabeled LHRH analogs as competitors was determined. Our results show that LHRH agonistic analog [D-Lys^6^]LHRH (as carrier peptide of AN-152), LHRH antagonist Cetrorelix and cytotoxic LHRH analog AN-152 could effectively bind to LHRH receptors at low nanomolar concentration (Table 4, Figure 4). Nevertheless, the cytotoxic LHRH conjugate AN-152 had only slightly lower binding affinity to specific LHRH receptors than the free peptide carrier [D-Lys^6^]LHRH (Table 4, Figure 4). Our results demonstrate that the high binding affinity of the carrier peptide [D-Lys^6^]LHRH to tumoral LHRH receptors are fully preserved in cytotoxic analog of LHRH, AN-152.

## 3. Discussion

More than 90% of urothelial tumors originate from the urinary bladder, 8% from the renal pelvis, and 2% from the ureter and urethra. Histologically, the majority (around 90%) of bladder cancer is described as the type of TCC and the rest are squamous cell carcinoma, adenocarcinoma, and rarely, small cell carcinoma [29]. Despite advancements in surgical techniques and chemotherapy, the overall survival in metastatic bladder cancer has not improved over the last 20 years [2,8,30]. The first-line therapy is cisplatin-based chemotherapy with the response rate of approximately 50% [4,31]. Unfortunately, about 30%–50% of the patients are unsuitable for cisplatin therapy, and there is no standard of care for this patient population. There is also no guideline for second-line treatment. This area remains an active field for oncology research [8,32].

In order to improve the prognosis of bladder cancer, early detection is of paramount importance. Standard prognostic features, such as pathologic stage and grade, have limited ability to predict the outcome for these patients; they do not adequately capture the individual biological potential and clinical behavior of the tumor to the therapy. Therefore, it is important to understand the molecular biological events that can explain and predict the clinical heterogeneity and behavior in response to the therapy of these lethal tumors. Molecular biomarkers, like cell-surface receptor expression are predictive for positive outcomes in targeted therapy [14,30].

Although there are numerous studies on benign and malignant lesions of the bladder, only a few were directed at hormones in the bladder. It has been shown that androgen receptors and estrogen receptors are expressed in the normal urothelium and in urothelial carcinoma, however their role is not fully understood, and most of the data have come from animal studies [33]. Androgens may contribute to the increased incidence of bladder cancer among men, while estrogens may exert a protective effect in women [34,35]. Studies with the lower urinary tract of intact male and female dogs showed LH receptor expression. LH receptor was present in all four regions and in three tissue layers of male and female dogs [36]. Bahk et al. (2008) detected mRNA expression for LHRH and LHRH receptors in four bladder cancer cell lines and five human bladder cancer tissues by using in situ hybridization [24]. It has been found that bladder cancer cell lines and human bladder mucosa tissue stained positively for LHRH or LHRH receptor by immunohistochemistry. Nevertheless, it could not be determined exactly whether both the LHRH and LHRH receptor are produced by the same cells or not [24]. Previously Szepeshazi et al. have shown the presence of LHRH receptors in surgical samples of human primary urothelial cancer by immunohistochemistry. The expression of LHRH-Rs was also demonstrated by Western blotting and binding assays in four human urinary bladder cancer cell lines (HT-1376, J82, RT-4 and HT-1197) [24]. However, no role has yet been found for extra-hypothalamic LHRH on bladder mucosa epithelium, especially in cancer.

In the present study, we have investigated the expression of mRNA for LHRH receptors in 24 bladder tumor specimens. In addition, to detect LHRH receptor in bladder cancer tissues, RT-112, UMUC3 and TCCSUP cell lines were also analyzed for receptor mRNA expression using RT-PCR method. Bladder cancer tissue specimens originated mostly from men (15/24) and, in a lesser occurrence, from women (9/24) with the diagnosis of bladder cancer, histologically differentiated as transitional cell carcinoma. PCR products for LHRH ligand were found in 19 of 24 (79%) specimens and mRNA for LHRH receptors was detected also in 20 of 24 specimens (83%). In 15 samples (62.5%), mRNA was detected for both LHRH and LHRH-R-I receptors. Seven female patients were positive for LHRH-R-I and five female patients were positive for LHRH-I ligand. Furthermore 11 male patients showed positive expression for LHRH-R-I and 13 male patients were positive for LHRH-I. The expression of mRNA for LHRH-R-I and LHRH ligand was also found in three bladder cancer cell lines investigated. These results might suggest that LHRH may function as an autocrine growth factor.

Our findings are in agreement with the study of Szepeshazi et al. [24]. In our study, the receptor protein was also examined by immunohistochemistry in 12 surgically removed human bladder specimens. Positive staining for LHRH receptor was found in nearly all of the samples examined, showing negative correlation with TCC grade. Localization of the LHRH receptor was observed in the form of brown stained dots in the cytoplasma or membrane of bladder cancer cells. In the study of Szepeshazi et al., positive staining for LHRH receptors was observed in all 18 human primary bladder cancer specimens examined. Enhanced staining of the plasma membrane as well as cytoplasmic staining were detected in malignant cells of urothelial bladder cancer [24]. In our study, on the basis of microscopic observation, the less differentiated TCC cases (Grade 3–4) showed no or weak staining, but the well or moderately differentiated tumors (Grade 1–2) showed moderate to strong LHRH receptor expression. Results of the Pearson analysis showed that the expression of LHRH receptors is negatively correlated (r = −0.91) with the pathological grade of the cases examined. Potentially only the low grade (Grade 1–2) patients expressing high enough levels of LHRH-R could be effectively targeted. Higher grade (Grade 3) patients or metastatic cases with lower expression of LHRH-R may not be suitable candidates for such targeted therapy using cytotoxic LHRH analogs. However, further studies are needed to clarify this question. In general, the positive immunostaining of LHRH receptor in bladder specimens was not related to the patients’ age or other clinical and/or pathological findings. The lack of receptor protein in some specimens may be related to the damage caused by transurethral resection (TUR) from which some of our samples were obtained. Using ligand competition assays, we also demonstrated the presence of specific, high affinity receptors for LHRH. It is important to note that all receptor positive human bladder cancer specimens examined by ligand competition assay and immunohistochemistry expressed a well-detectable amount of the LHRH-R-I gene. The expression of LHRH-R protein was also confirmed by Western blot analysis in the three cell lines investigated.

Our findings are in agreement with the results of Bahk et al. (2008) [24]. We were able to demonstrate that a high percentage of human bladder cancer specimens obtained from surgery expresses LHRH and its receptors. However, there is no information about the role of peptide receptors in bladder carcinogenesis. The signaling pathways have the potential to be an important area of bladder cancer research, leading to the development of effective therapeutic approaches [15,32]. The high incidence of LHRH and LHRH receptors in human bladder cancer suggests that hormones and related receptors may play a role in the regulation of tumor cell growth and function. The presence of both the ligand and the receptor indicates that LHRH could have a paracrine or autocrine effect, although we do not know the exact mechanism. Bladder cancer represents a considerable opportunity and challenge for molecularly targeted therapy. Potent antagonists of LHRH, such as Cetrorelix, Ganirelix, Abarelix, and Degarelix, have been developed and are now available for clinical use in gynecology and oncology [12]. The receptors for LHRH on human tumors can also serve as targets for LHRH analogs linked to cytotoxic agents. The cytotoxic analog AN-152 (AEZS-108, zoptarelin doxorubicin), consisting of [D-Lys^6^]LHRH linked through a glutaric acid spacer to doxorubicin (DOX) was designed for receptor mediated chemotherapy aimed at the inhibition of the growth of tumors expressing LHRH receptors [26]. Szepeshazi et al. showed that AEZS-108 inhibits powerfully the growth of the bladder cancer in nude mice and exerted greater effects than DOX with lesser toxicity [12]. This analog has also been extensively investigated in a large number of human experimental studies and tested in Phase I/II clinical trials in castration-resistant prostate, breast and metastatic bladder cancers, and in Phase II and III trials in endometrial and ovarian cancer [15]. As discussed in a review paper by Engel et al., because of the very promising Phase II results in endometrial cancer, a multinational, multicenter Phase III study of this malignancy has also been initiated [37,38]. Based on preclinical and clinical results AEZS-108 seems to be a promising candidate for targeted therapy for the patients with LHRH-R positive cancer [37,38]. Regrettably, clinical trials with zoptarelin doxorubicin in various human cancers were already either completed or terminated, because some of these studies did not achieve their primary endpoint [37].

Our results support the merit of additional investigation of the role of LHRH and its receptors in human bladder cancer and open up a new avenue in the further development of LHRH analogs for therapeutic and imaging purposes in bladder cancer. In the future, we would like to expand our investigation and we try to collect a reasonable number of healthy and tumorous bladder samples to further study the expression of LHRH-Rs in such human tissues. These studies may provide novel quantitative data about the mRNA and protein levels of LHRH receptors. From these results we would be able to predict the potential response of the patients to LHRH-R-based therapy.

## 4. Materials and Methods

### 4.1. Bladder Cancer Tissue Samples

A total of 24 surgically resected bladder cancer tissues was used in the study (mean age, 65 years; range: 42–88 years) (Table 2). The specimens were obtained at the time of initial open surgical treatment from at the Department of Urology, University of Debrecen, Hungary. The collection and the use of these specimens and normal human pituitary samples in our studies was conducted in accordance with the Declaration of Helsinki and approved by the local institutional ethics committee named Regional Institutional Ethics Committee, Clinical Center, University of Debrecen (UD REC/IEC 4831-2017). Informed consent was obtained from all patients. Immediately after surgical removal, a small portion of the bladder tissues was collected, saved and snap frozen in liquid nitrogen, and stored at −80 °C until molecular biology analyses. For histopathological analysis formalin-fixed paraffin embedded tissues were used. Normal human pituitary reference samples used as positive controls were collected from the paraffin tissue-archives of autopsy cases in an anonymous fashion at the Department of Pathology, University of Debrecen.

### 4.2. Histology

Surgically resected, formalin-fixed paraffin-embedded tissues of bladder (91.7% transitional cell carcinoma: TCC) from 24 patients were analyzed. Sections with the thickness of 2–3 µm were routinely stained with hematoxylin–eosin (HE) standard method [39,40,41,42]. TCC tissue sections were evaluated in accordance with the recent guidelines for TCC grading to confirm the original diagnosis. Representative histology images for the applied grading results are shown in Supplementary Materials Figure S1. None of the patients received chemotherapy or radiotherapy prior to the surgery. The clinicopathological findings of the patients are shown in Table 2.

### 4.3. Cell Culturing

Human bladder cancer cell lines RT-112, UMUC3, and TCCSUP were kind gifts of Professor Roman Blaheta (Goethe University, Frankfurt, Germany). Bladder carcinoma cells were cultured in RPMI 1640 supplemented with 10% fetal bovine serum (FBS), 1% penicillin/streptomycin (Gibco/Invitrogen, Darmstadt, Germany) in a humidified, 5% CO_2_ incubator. RT-112 is an invasive (pathological stage T2) moderately differentiated (grade 2/3) model of human bladder cancer, UMUC3 is a high grade 3 invasive bladder cancer, and TCCSUP represents a grade 4 transitional cell carcinoma.

### 4.4. Isolation of RNA

Approximately 30 mg of each surgically removed tissue sample was homogenized using an Ultra-Turrax tissue homogenizer (IKA Labortechnik, Staufen, Germany). Total RNA was extracted using Nucleospin Total RNA and Protein Isolation Kit (Macherey-Nagel, Düren, Germany) according to the manufacturer’s instructions. The isolated RNA was resuspended in RNase free water and kept at −80 °C until further use. RNA concentration and purity were determined using the Nanondrop ND-1000 UV Spectrophotometer (Nanodrop Technologies, Wilmington, DE, USA). The integrity of total RNA was verified by gel electrophoresis.

### 4.5. RT-PCR

For reverse transcription, 150–250 ng of total RNA, oligo (dT) 15 primer, and MMLV Reverse Transcriptase (Promega Corporation, Madison, WI, USA) were used according to the manufacturer’s instructions. First-strand cDNA was amplified using gene-specific primers for LHRH-R-I: sense 5′-GAC CTT GTC TGG AAA GAT CC-3′, antisense 5′-CAG GCT GAT CAC CAC CAT CA-3′, for LHRH-I sense: 5′-CTA CTG ACT TGG TGC GTG GA-3′ and antisense: 5′-CTG CCC AGT TTC CTC TTC AA-3′. For β-actin houskeeping gene, sense 5′- GGC ATC CTC ACC CTG AAG TA-3′, and antisense 5′-GGG GTG TTG AAG GTC TCA AA-3′ were used [37]. After reverse transcription (RT) reaction, 1 µL cDNA of each sample, was subjected to PCR amplification. The reaction mixture contained 1 µL cDNA, 1.5 mM MgCl_2_, 0.5 µM of each primer (Invitrogen, Birmingham, UK), 1× PCR buffer, 200 µM of each dNTP, and 1U Taq Polymerase (Invitrogen, Birmingham, UK) in a final volume of 25 µL. After denaturation (3 min at 94 °C) cDNA was amplified for 45 cycles (45 s at 94 °C; 30 s at 60 °C; and 1 min 30 s at 72 °C). For β-actin amplification, the whole PCR reaction involved 30 cycles. A final elongation step at 72 °C for 10 min was then applied (β-actin amplification was performed in order to test the quality of cDNA samples used for further RT-PCR analyses). PCR products were separated electrophoretically in 1.5% agarose gel and stained with GelRed^®^ Nucleic Acid Gel Stain (Biotium, Cambridge, UK). To avoid genomic DNA contamination, samples were treated with DNase before RT-PCR.

### 4.6. Immunohistochemistry

For immunohistochemistry (IHC), serial sections of paraffin-embedded tissues (12 samples) were used as described previously using the immuno-peroxidase technique [21,22]. Briefly, after dewaxing in xylene and rehydrating in graded alcohol, antigen retrieval was performed in a pressure cooker for 3 min in citrate buffer (0.1 M pH 6.0; Target Retrieval Solution, Dako, Glostrup, Denmark). For biotin-free immune staining, a ’Bond-TM Automated Immune-Stainer’ (Leica Microsystems, Newcastle, UK) was used according to the manufacturer’s instruction (NovoCastra Visionbiosystems Bond TM, Novocastra Laboratories Ltd., Newcastle, UK). As a primary antibody, GnRH-R (equivalent to LHRH-R-I) monoclonal antibody (mAB; NCL-GnRHR A9E4; Novocastra Laboratories Ltd., Newcastle, UK) was applied in 1:20 dilution. Slides were incubated for 30 min with NCL-GnRHR mAB targeting the human GnRH terminal region. The immunoreaction was visualized by using diamino-benzidine (DAB). The slides were then counterstained with Mayer’s hematoxylin. Immunoreactions were analyzed using a Leica-DM2500 microscope equipped with a 12 Mpixel digital camera (Leica-Microsystems, Newcastle, UK) and semiquantitatively evaluated by a pathologist with respect to the intensity of the immunoreaction using the following scoring system: 0, no staining; 1+, weak; 2+, moderate; 3+, strong staining (Figure S2). Human pituitary glands (anterior lobe) obtained from archives of the autopsy cases with no tumor or metabolic disorder were used as positive controls (Figure S3). To check the staining specificity, negative controls were also included in each immunohistochemistry run where the primary antibody was replaced with species specific normal serum in place of primary antibody. Occasionally, the LHRH antibody was preabsorbed to the LHRH protein and, in turn, the incubation with the tissue section was then applied with the cocktail (ab-antigen) proteins instead of the primary alone. In both cases, no stainings were found.

### 4.7. LHRH Receptor Binding Studies

Preparation of tumor membrane fractions for LHRH receptor binding studies was performed as described previously [17,22]. Receptors for LHRH on human bladder cancer specimens were characterized by ligand competition assays based on binding of [^125^I][D-Trp^6^]LHRH as a radioligand to membrane homogenates of bladder cancer [17,22]. This radioligand was well-characterized previously and showed high affinity binding to LHRH receptors expressed in various human cancers [17,18,22]. The binding potencies of LHRH analogs and cytotoxic LHRH analog AN-152 to LHRH receptors were determined by displacement of [^125^I][D-Trp^6^]LHRH binding. The assay was performed as described earlier [17,26]. In brief, membrane homogenates of bladder cancer specimens containing 60–150 µg protein were incubated in duplicate with 70–90,000 cpm [^125^I][D-Trp^6^]LHRH in the presence of increasing concentrations (10^−12^–10^−6^ M) of nonradioactive unlabeled peptides as competitors, in a total volume of 150 µL of binding buffer. At the end of the incubation, 125 µL aliquots of suspension were transferred onto the top of 1 mL of ice-cold binding buffer containing 1.5% BSA in microcentrifuge tubes. The tubes were centrifuged at 12,000× *g* for 3 min at 4 °C. Supernatants were aspirated and the pellet was counted in a gamma counter. Protein concentration was determined by the method of Bradford using a Bio-Rad protein assay kit (Bio-Rad Laboratories, Hercules, CA, USA). The final binding affinities were expressed as IC_50_ values and were calculated by using the LIGAND-PC computerized curve-fitting program of Munson and Rodbard [17,26].

### 4.8. Western Blot Analysis

For cell lysate preparation, RT-112, UMUC3, and TCCSUP bladder cancer cells were washed with ice-cold phosphate-buffered saline (PBS). Afterwards, the cells were scraped from the flasks and lysed in ice-cold M-PER protein lysis buffer (Thermo Fisher Scientific, Waltham, MA, USA), supplemented with protease and phosphatase inhibitors (Sigma-Aldrich, St. Louis, MO, USA). Protein quantification of cell lysate was performed using BCA reagent (Thermo Fisher Scientific, Waltham, MA, USA). All the samples were diluted with 4× Laemmli buffer. Equal amount (40 μg) in equal volume of each protein sample was boiled at 95 °C for 8 min. Corresponding to the molecular weight of the target protein, the lysates were separated on 10% sodium dodecyl sulfate–polyacrylamide gel by electrophoresis (SDS-PAGE). As a molecular weight marker Precision Plus Protein Kaleidoscope Standard (BioRad Laboratories, Irvine, CA, USA) was used (Figure S4). The proteins were transferred to polyvinylidene fluoride (PVDF) membrane (Millipore, Burlington, MA, USA). Upon blocking with 5% milk-TBS-Tween, membranes were incubated with primary antibodies (overnight, 4 °C): anti-LHRH-R, 1:200 dilution (sc-13944 rabbit polyclonal; Santa Cruz Biotechnology Inc., Dallas, TX, USA), anti-GAPDH, 1:1000 dilution (D16H11 rabbit monoclonal; Cell Signaling Technology, Danvers, MA, USA). Incubation with primary antibodies was followed by HRP-tagged anti-mouse IgG secondary antibody (Thermo Fisher Scientific, Waltham, MA, USA). The signal was detected by chemiluminescence. Intensity of each band was normalized to GAPDH.

## 5. Conclusions

In summary, our results provide evidence about the expression of LHRH and LHRHR-I in human bladder cancer tissues and human bladder cancer cell lines. The high incidence of LHRH-R in BC suggests that it could serve as a molecular target for therapy of human BC with cytotoxic LHRH analogs or modern powerful antagonistic analogs of LHRH. Our findings may contribute to further development of LHRH analogs for potential therapeutic application and imaging purposes in human bladder cancer.

## Figures and Tables

**Table 1:** Expression pattern of mRNA for LHRH-I and LHRH-R-I in human bladder cancer specimens.

	Positive/Total Number of Cases Examined	Positive (%)
**LHRH-I mRNA**	19/24	79
**LHRH-R-I mRNA**	20/24	83

**Table 2:** Clinicopathological findings, mRNA expression pattern of LHRH-I and LHRH-R-I; LHRH receptor protein expression in human bladder cancer specimens.

Number	Age/Sex	Clinicopathological Findings	Grade	Stage	LHRH-I	LHRH-R-I	IHC Results
1	43/female	transitional cell carcinoma	G3	pT2	−	+	1+
2	69/male	transitional cell carcinoma	G1-2	pT1	+	−	N/A
3	56/male	transitional cell carcinoma	G3	pT3	+	+	1+
4	63/female	transitional cell carcinoma	G1	N/A	+	−	N/A
5	89/female	transitional cell carcinoma	G3	pT2	+	+	1+
6	51/male	transitional cell carcinoma	G1-2	pT1	+	+	2+
7	66/male	transitional cell carcinoma	G2	pT2, pNx, pMx	+	+	2+
8	67/male	transitional cell carcinoma	G3	pT3a, pNx, pMx	+	+	1+
9	69/male	transitional cell carcinoma	G1	pT1, pNx, pMx	+	+	2+
10	50/male	transitional cell carcinoma	G1	pT1, pNx, pMx	+	+	N/A
11	68/male	transitional cell carcinoma	G1	pT1, pNx, pMx	+	+	N/A
12	42/female	transitional cell carcinoma	G2-3	N/A	−	+	N/A
13	79/male	transitional cell carcinoma	G1-2	pT1	+	+	2+
14	84/female	transitional cell carcinoma	G1	N/A	+	+	N/A
15	63/male	transitional cell carcinoma	G2	N/A	+	+	2+
16	70/female	transitional cell carcinoma	G3	pT2	+	−	N/A
17	63/female	transitional cell carcinoma	G2	pT1	−	+	N/A
18	51/female	transitional cell carcinoma	G1	N/A	−	+	3+
19	71/female	transitional cell carcinoma	G3	pT2	+	+	1+
20	86/male	transitional cell carcinoma	G2	pT1	+	+	N/A
21	57/male	transitional cell carcinoma	G1	N/A	+	+	N/A
22	50/male	transitional cell carcinoma	G3	pT3	−	+	1+
23	84/male	transitional cell carcinoma	G2	pT1	+	−	N/A
24	74/male	transitional cell carcinoma	G3	pT2	+	+	N/A

**Table 3:** Expression of mRNA and binding characteristics of LHRH-R in 12 human bladder cancer specimens.

Patient Number	LHRH-R mRNA	Kd (nM)	Bmax (fmol/mg (Protein))
3	+	5.76	274.5
4	−	−	−
6	+	6.85	273.1
7	+	4.94	255.0
8	+	6.26	685.2
10	+	5.02	721.4
12	+	3.61	704.5
14	+	4.92	578.9
16	−	−	−
18	+	4.38	445.3
19	+	3.83	421.6
22	+	4.31	371.4

Kd: dissociation constant; Bmax: maximal binding capacity; values were calculated from duplicate tubes.

**Table 4:** Receptor binding potency (IC_50_ values) of LHRH analogs to the membrane receptors of human bladder cancer cells.

Compound	IC_50_ Values (nM)
[D-Lys^6^]LHRH	0.98
Cetrorelix	0.86
AN-152	1.39

IC_50_ values were calculated by computerized curve-fitting program from displacement experiments, as described earlier [17,18,22,26]. IC_50_ is defined as the dose causing 50% inhibition of specific binding of [^125^I][D-Trp^6^] LHRH to the membranes. Values are mean of two to three determinations.

## Data Availability

The data presented in this study are available on request from the corresponding author.

## Supplementary Materials

The following are available online, Figure S1: Representative transitional cell carcinoma (TCC) samples stained by HE, Figure S2: Representative images of LHRH-R protein on IHC labeled tissue sections of TCC bladder cancers, Figure S3: Representative positive control images for IHC obtained from a normal human pituitary gland, Figure S4: Chemiluminescence detection of LHRH-receptor protein in human bladder cancer cell lines

## Sample Availability

Not available.
