# Supplementary material for: Expression of Luteinizing Hormone-Releasing Hormone (LHRH) and Type-I LHRH Receptor in Transitional Cell Carcinoma Type of Human Bladder Cancer
Source: Molecules. 2021 Feb 26;26(5):1253. doi: 10.3390/molecules26051253 (PMC7956722; doi:10.3390/molecules26051253)
Supplement: Supplementary file 1 [file molecules-26-01253-s001.zip › Szabo et al Figure S1.pdf]

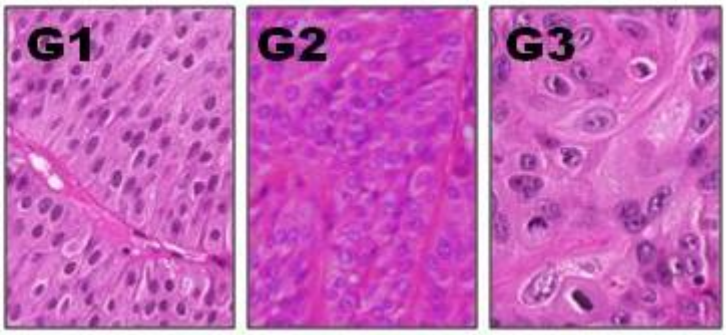

Figure S1. Representative transitional cell carcinoma (TCC) samples stained by HE. Representative samples stained by HE demonstrate the grading of transitional cell carcinoma (TCC) according to the tumor architecture, the cytologic and nuclear morphologies of the neoplastic cells. **G1** corresponds to a low grade (non-invasive) TCC; **G2** can be either low grade or high grade tumor (the latter is shown on this image). **G3** is equivalent to high grade TCCs (the demonstrated images are from invasive neoplasms except the left image for G1). Magnifications for all images: 250x. The differentiation levels of urothelial carcinomas were evaluated based on the guidelines (Ref 39-42).
