# Supplementary material for: Expression of Luteinizing Hormone-Releasing Hormone (LHRH) and Type-I LHRH Receptor in Transitional Cell Carcinoma Type of Human Bladder Cancer
Source: Molecules. 2021 Feb 26;26(5):1253. doi: 10.3390/molecules26051253 (PMC7956722; doi:10.3390/molecules26051253)
Supplement: Supplementary file 1 [file molecules-26-01253-s001.zip › Szabo et al Figure S2.pdf]

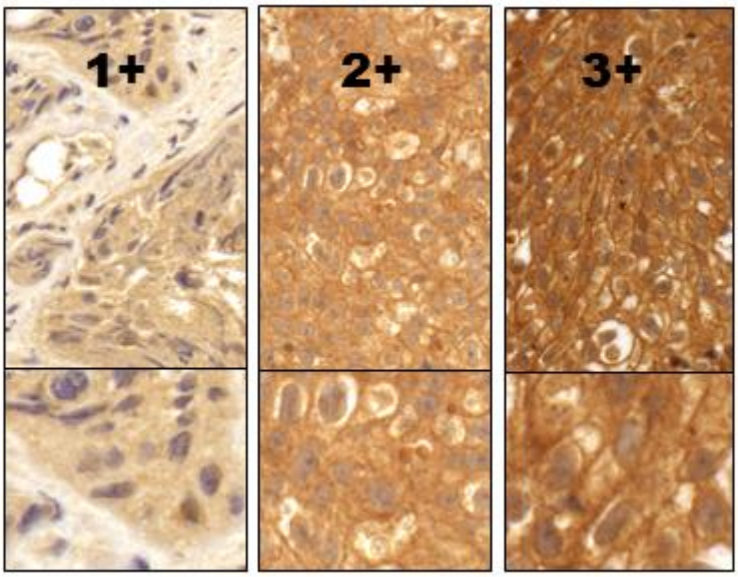

Figure S2. Representative images of LHRH-R protein on IHC labeled tissue sections of TCC bladder cancers. Representative images of LHRH-R protein on IHC labeled tissue sections harbouring TCC bladder cancers are shown to demonstrate the semiquantitative scoring for LHRH-Rs expressed by neoplastic cells. **1+** demonstrates a high grade (G3) invasive TCC case where minimal tumor cell staining is seen; **2+** shows a high grade (G3) invasive TCC where moderate expression is observed; **3+** is a case of low grade (G2) papillary transitional cell carcinoma of non-invasive type where intense positive staining is detected, both with cytoplasmic and membranous patterns. The lower panel images are to demonstate the cellular details in high magnifications. Magnification for upper panel: 250x, and 400x for lower panel, respectively. To check the specificity of the LHRH-R antibody on tissue sections, in each IHC run, a negative control slide was also included where isotype-specific normal immunoglobulin was used in place of the primary antibody which did not reveal positive staining.
