# Supplementary material for: Expression of Luteinizing Hormone-Releasing Hormone (LHRH) and Type-I LHRH Receptor in Transitional Cell Carcinoma Type of Human Bladder Cancer
Source: Molecules. 2021 Feb 26;26(5):1253. doi: 10.3390/molecules26051253 (PMC7956722; doi:10.3390/molecules26051253)
Supplement: Supplementary file 1 [file molecules-26-01253-s001.zip › Szabo et al Figure S3.pdf]

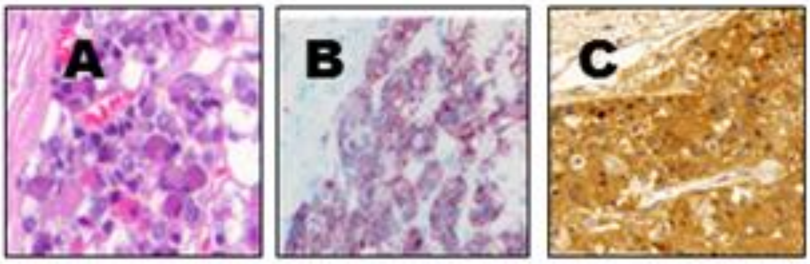

Figure S3. Representative positive control images for IHC obtained from a normal human pituitary gland. Representative positive control images for IHC obtained from a normal human pituitary stained with HE. **(A)** The endocrine cells of the adeno-hypohysis region (right) are typically expressed the common marker synaptophysin (**B)** purple cells using VIP chromogen) and LHRH-R protein **(C)** brown cells using DAB chromogen, respectively while the neuro-hypophysis parts (left) are non-reactive for these proteins. Magnifications for all images: 150x. To check the specificities for the primary antibodies, each IHC reaction included negative control where the primary antibody was replaced with an isotype-specific normal immunoglobulin which did not give positive staining.
