# Supplementary material for: Expression of Luteinizing Hormone-Releasing Hormone (LHRH) and Type-I LHRH Receptor in Transitional Cell Carcinoma Type of Human Bladder Cancer
Source: Molecules. 2021 Feb 26;26(5):1253. doi: 10.3390/molecules26051253 (PMC7956722; doi:10.3390/molecules26051253)
Supplement: Supplementary file 1 [file molecules-26-01253-s001.zip › Szabo et al Figure S4.pdf]

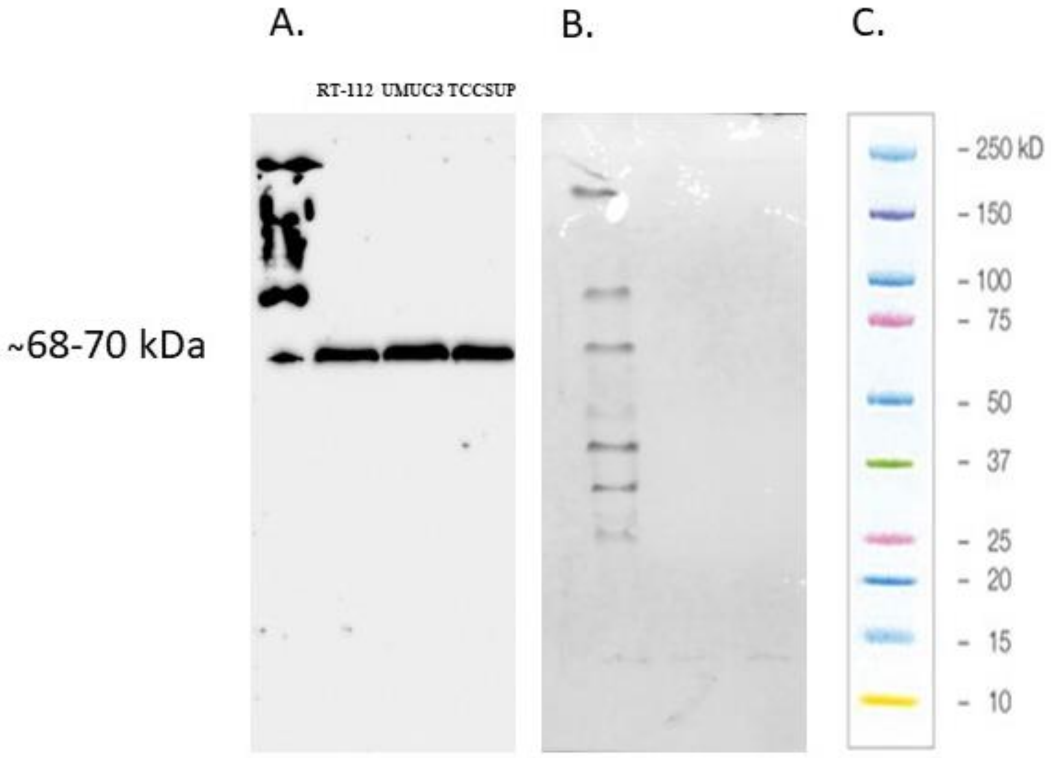

Figure S4. Chemiluminescence detection of LHRH-receptor protein in human bladder cancer cell lines. (A) Chemiluminescence detection of LHRH-receptor protein in human bladder cancer cell lines (RT-112, UMUC3, TCCSUP). (**B)** Image of membrane with visualized marker used for 10 %SDS –PAGE (Precision Plus Protein™ Kaleidoscope™ Prestained Protein Standards #1610375). (**C)** Picture of Kaleidoscope standard # 1610375 provided by the manufacturer (BIORAD).
